# A PNU-Based Methodology to Improve the Reliability of Biometric Systems

**DOI:** 10.3390/s22166074

**Published:** 2022-08-14

**Authors:** Paola Capasso, Lucia Cimmino, Andrea F. Abate, Andrea Bruno, Giuseppe Cattaneo

**Affiliations:** Department of Computer Science, University of Salerno, 84084 Fisciano, Salerno, Italy

**Keywords:** biometric, face recognition, PNU, Source Camera Identification, spoofing

## Abstract

Face recognition is an important application of pattern recognition and image analysis in biometric security systems. The COVID-19 outbreak has introduced several issues that can negatively affect the reliability of the facial recognition systems currently available: on the one hand, wearing a face mask/covering has led to growth in failure cases, while on the other, the restrictions on direct contact between people can prevent any biometric data being acquired in controlled environments. To effectively address these issues, we designed a hybrid methodology that improves the reliability of facial recognition systems. A well-known Source Camera Identification (SCI) technique, based on Pixel Non-Uniformity (PNU), was applied to analyze the integrity of the input video stream as well as to detect any tampered/fake frames. To examine the behavior of this methodology in real-life use cases, we implemented a prototype that showed two novel properties compared to the current state-of-the-art of biometric systems: (a) high accuracy even when subjects are wearing a face mask; (b) whenever the input video is produced by deep fake techniques (replacing the face of the main subject) the system can recognize that it has been altered providing more than one alert message. This methodology proved not only to be simultaneously more robust to mask induced occlusions but also even more reliable in preventing forgery attacks on the input video stream.

## 1. Introduction

Face recognition is one of the most active applications of pattern recognition, image analysis, and understanding [1], with it having become one of the most widely biometric solutions in several security systems and real-world scenarios. Modern systems for crime prevention, law enforcement applications, access control, and surveillance, such as Intelligent Closed-Circuit Television (ICCTV) or IPV4 cameras systems, have attracted a great deal of attention. These systems capture images from a CCTV/IP camera and apply a face recognition algorithm to identify the detected subject and grant access to a specific gate or restricted area. If the subject’s identity is verified as permitted, access is allowed; otherwise, it is denied.

When these systems are used in sensitive areas (i.e., airports, military zone, etc.), the integrity of the data used in the acquisition phase, together with the accuracy of the biometric recognition, becomes a critical aspect of the security of the system.

The COVID-19 outbreak has created a series of new problems that are negatively impacting the reliability of the previous generation facial recognition systems designed for people who did not have anything covering their faces. On one hand, the wearing of a facial mask drastically reduces facial recognition performances, since almost half of the face is covered and no longer available for acquisition [2]. On the other, the restrictions on travel and contact between people to limit the virus spreading can make the acquisition of biometric data in a controlled environment complicated, if at times impossible. Masked Face Recognition (MFR) has gained more and more attention over the past two years. Due to the loss of crucial information relating to the parts of the face such as the chin, cheeks, lips, and nose, the facial recognition software used at various checkpoints [3] performs relatively badly when there are masked faces. For authentication and verification applications such as for unlocking phones, making digital payments, and carrying out public safety inspections, among others, the failure of facial recognition methods in the case of face masks has created significant challenges [4,5]. For example, the entry gates at public security checkpoints at train and bus terminals have installed cameras that rely on a face recognition technology that is unable to identify people wearing face masks/coverings [6]. Current research is now focusing on the area of face mask spoofing as a result of the rise in fraud attempts in this new real context. Additional characteristics must be introduced to make the biometric system safer and computationally efficient against sneaky and unpredictably spoofing attacks.

This work proposes a facial recognition system that uses facial dynamics to effectively recognize a subject, with and without a mask or face covering, in which the images acquired for the enrollment phase are subjected to a forgery detection phase before accepting their authenticity.

Face masks hide a significant part of the human face and make several facial features unavailable for analysis. The applied recognition system aims to analyze the dynamics of the periocular feature to increase recognition accuracy. A deep learning approach is used to learn from the temporal features. A Convolutional Neural Network–Long Short-Term Memory (CNN-LSTM) architecture is applied to the video sequences to use the facial dynamics for the classification process, with only the periocular region (the area not covered by a face mask) being taken into consideration.

In a context where there is an urgent need for a high level of security, it was possible to introduce a further check on the integrity of the data acquired. We introduced an identification technique, mostly based on the Pixel Non-Uniformity noise (PNU noise, for short), which is widely used in current literature to automatically examine images under investigation. PNU is a noise characteristic of digital camera sensors, and it is extremely efficient when used for identification tasks, such as those performed by monitoring systems, as well as to prevent fraud scams. Due to its effectiveness and reliability, its use has now been extended to other relevant application domains such as biometric recognition to resolve the integrity of their authentication systems.

To make the facial recognition system both more robust as well as more reliable even in the case of counterfeiting operations, in combination with a facial recognition system that can recognize masked faces with a greater accuracy, we have included an additional level of security through the PNU-based Source Camera Identification (SCI, for short) approach, which allows evaluating the integrity of video images.

In this biometric system, before or during feature extraction for the machine learning approach, we introduced the SCI method to identify and possibly exclude those videos whose frames have been tampered with or which, in any case, have a low level of reliability.

According to the hybrid technique described above, by exploiting the reliability and accuracy of the PNU-based SCI approach, it is possible to make a more robust facial recognition system when a subject is wearing a face mask or in the presence of attempts, and in general, any biometric system.

The rest of the paper is organized as follows. In Section 2, we describe the aim of this study. Section 3 discusses the current state of the art in face recognition systems. Section 4 and Section 5 present the experimental analysis. Section 6 discusses the results of the face recognition procedure in combination with the PNU-based SCI method. Finally, Section 7 presents some conclusions.

## 2. The Face Recognition Problem

In recent years, facial recognition has become a particularly relevant topic, reaching a relatively high level of accuracy under conditions where there is high variability in the pose, expression, in terms of face size, ambient lighting, and distance from the camera. The use of face masks/coverings represents a new factor, which can often negatively affect the recognition process.

This has drawn attention to the consequent threats and vulnerabilities that reduce the reliability standard of face recognition and security systems. To improve the performance of standard face recognition systems and identity verification, a fusion between hard and soft biometrics is often applied [7,8,9].

This study considers the spatial and temporal features for face recognition in presence of face masks/coverings. Consequently, the dataset includes video acquisitions of the same subjects with and without a face mask. All the images, extracted from a single video, were collected in a fixed number of partitions and referable to a subject with and without a mask. Therefore the difference in performance cannot be attributed to changes in the camera unless there has been tampering during the image acquisition phase. The SCI method works on precisely this point. Any changes in the pose are not a determining factor since the datasets are made up of frontal images. Conversely, a potential source of the variability of the performance is given by the fact that people, regardless of whether they are wearing a mask or not, vary their “recognizability”. This is where face recognition techniques play a decisive role.

A biometric recognition system uses physical characteristics (e.g., fingerprints, iris, face, ear, etc.) and/or behavioral characteristics (e.g., voice, signature, handwriting, etc.) for the identification or recognition of a subject. Facial recognition addresses the problem of identifying and/or authenticating a person from a photo or video using the human face as the main biometric feature. It has also recently attracted attention in developing deep learning methods, with the main advantage being that no pre-processing is required. The network is fed directly with the images avoiding the features extraction phase. Convolutional neural networks, or ConvNet (CNN), are one of the most widely used deep learning algorithms in computer vision and are currently the most performing facial recognition methods for image classification and facial recognition activities [10,11]. The most sophisticated models that use deep learning in multiple time domains complement the Convolutional Neural Networks with the use of Recurrent Neural Network (RNN). For example, to combine static and dynamic characteristics in a CNN-RNN framework, a Long Short-Term Memory Network could be combined with convolutional layers. Long Short-Term memory (LSTM) is an artificial recurrent neural network (RNN) in which connections between the nodes form a direct or undirected graph along a timeline that allows them to exhibit a dynamic temporal behavior [12].

### 2.1. The Source Camera Identification (SCI) Approach

The SCI problem concerns the identification of the digital camera used to acquire the digital images and their integrity [13]. Current literature highlights how each digital camera has its own unique Pixel Non-Uniformity Noise (PNU noise, for short), i.e., is the characteristic noise left by the digital camera sensor when capturing images. The latter could be used as a kind of fingerprint for identification activities.

There are multiple contributions to SCI methods and one of the most reliable and robust SCI techniques proposed so far to extract noise (PNU) from a digital camera sensor is [14] by Fridrich et al. The other contributions in line with the SCI methods are essentially a variant of this algorithm.

There is a brief overview of this algorithm, since it used as a reference method in our experiment.

#### 2.1.1. The Fridrich et al. SCI Procedure

Let *I* be a digital image under investigation and *C* a digital camera. We are interested in knowing whether *I* was probably taken using *C*.

First, extract the PNU noise from I and then correlate it with an estimation of the reference sensor pattern noise (Reference Pattern, RP) of C.

The identification made using the Fridrich et al. procedure can be summarized in the following three phases:

Phase 1

With the following formula, we extract a Residual Noise (RN) from *I*:

(1) $$RN_{I}=I-F\left(I\right)$$

where *F* is a denoising function, such as a Daubechies 8 low-pass filter wavelet. The extracted noise RN, denoted as $RN_{I}$, is a Pixel Non Uniformity (PNU) noise approximation existing in *I*, since the latter cannot be directly obtained (see [14] for details).

Phase 2

The PNU pattern RP of *C* is estimated from a collection *M* of images taken with the camera *C*. This can be done by averaging the RNs from a set of images taken using C as defined in (2):

(2) $$RP_{C}=\frac{\sum_{i=1}^{M}RN_{i}^{\left(C\right)}}{M}$$

Phase 3

To measure the similarity between $RN_{I}$ (i.e., the residual noise of *I*) and $RP_{C}$ (i.e., the PNU pattern of *C*) a statistical correlation is applied. This can be achieved by using one of several approaches such as the Circular Cross-Correlation Norm (CCN) (see [15]) statistic, the Peak-to-Correlation Energy (PCE) statistic (see [16]), and the Bravais-Pearson Correlation (CCBP) $\rho_{C}$ statistic (see [14]), used in this work and defined as follows:

(3) $$\rho_{C}\left(p\right)=corr\left(n,P_{C}\right)=\frac{\left(n-\bar{n}\right)\cdot \left(P_{C}-{\bar{P}}_{C}\right)}{\|\left(n-\bar{n}\right)\left\|\right\|\left(P_{C}-{\bar{P}}_{C}\right)\|}$$

where the identification occurs by comparing $\rho_{C}$(p) with a certain threshold θ, estimated according to the Neyman–Pearson method. If the value of this statistic exceeds θ, then it is probable that *I* was taken from *C*.

## 3. Face Recognition Systems

A typical video-based facial recognition system automatically detects face regions, extracts features from the video, and recognizes the face’s identity if a face is present. In surveillance, information security, and access control applications, facial recognition and identification from a video sequence is an important problem. The recognition of faces from video sequences is a direct extension of still-image-based recognition. Video-based face recognition techniques use both spatial and temporal information.

Video face recognition originated from still-image-based techniques. This kind of system automatically detects and segments the face from the video and then applies still-image face recognition techniques [17,18,19]. In [20], the authors apply a dual-input CNN to efficiently recognize the subjects only through the periocular area. The experimentation is carried out on a video dataset after the extraction of facial images from the video frames. Recently, video-based face recognition methods exploit both spatial information (in each frame) and temporal information (such as the trajectories of facial features) [21]. A big difference between these methods and the still-image-based techniques (i.e, [17]) is the use of representations in a joint temporal and spatial space for identification. To extract temporal characteristics from video sequences, Baccouche et al. [22] apply embedded LSTM units to features extracted with a Scale-Invariant Feature Transform (SIFT) algorithm. By applying this strategy to a CNN structure, a CNN-LSTM model is obtained, in which the LSTM units are cascaded to a CNN. Iengo et al. [23] evaluate how a set of geometrical features, defined as distances between landmarks placed in the bottom half of the face, changes over time when a sentence is spoken. In [24], the authors explore a dynamic approach for biometric face recognition, considering both the periocular and labial dynamics of the facial landmarks during the pronunciation of a sentence.

## 4. Our Contribution

With the COVID-19 pandemic and the difficulties of acquiring biometric data in controlled environments, new facial recognition problems have emerged that seriously endanger the reliability and security of the systems. As a result, several applications nowadays have reduced their recognition performance. In this work, the designed hybrid approach aims to improve access control systems based on face recognition. On the one hand, the proposed face recognition approach, based on facial dynamics, faces the problem of recognizing people wearing face masks/coverings. On the other, it cooperates with the PNU-based SCI technique to detect any tampered or unreliable frames belonging to the input video streams.

**Figure 1:**
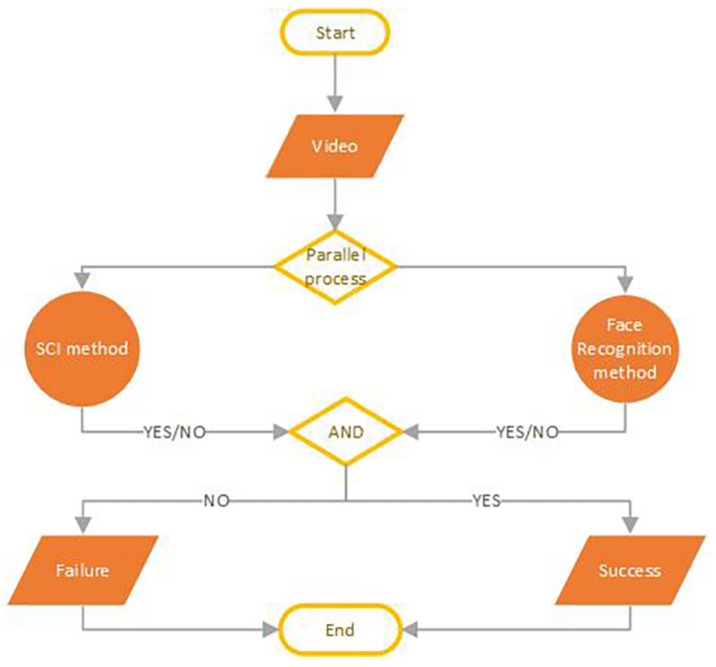
Flow-Chart of biometric face recognition system with Source Camera Identification (SCI). The facial recognition process and the SCI method work as long as both the procedures are successful. Otherwise, the general system fails.

According to this procedure, in a controlled environment during the recognition phase, the acquired video frames are subjected to a counterfeiting detection phase to validate their authenticity. If a subject is correctly identified through the facial recognition system and all the video frames are unmodified, then the permission/access is granted; otherwise, it is denied. The applied methodology is shown in Figure 1.

### 4.1. The SCI Procedure

It is nowadays possible to see specific real-world forensic scenarios, such as, for example, a video that has been tampered with, i.e., falsified by adding sequences of frames (alien frames) recorded using a different, unavailable device, or the camera is unavailable but there is only a counterfeit video, originally recorded with that camera but containing a variable percentage of alien frames [25].

Consequently, our attention is focused either on the problem of evaluating the integrity of a video file [26], threatened with spoofing [27] or on the compliance of a target source camera that was used to acquire the image (i.e., SCI) [14,28].

As noted in [29], digital video cameras use the same imaging sensors used by digital cameras; therefore, it is possible to extend, with high reliability, the most relevant PNU-based technique presented in [14] to detect forgeries in digital images and identify digital video cameras from video clips. Starting from these assumptions, we intend to apply the SCI algorithm as an anti-spoofing measure to support a biometric recognition system, such as a face, for instance, to certify the integrity of the image. It is therefore possible to avoid criminal acts and fraudulent activities through a distributed system with a high temporal performance.

### 4.2. The Face Recognition Procedure

Face recognition has recently gained a lot of attention as one of the most successful applications in the computer vision field, especially in recent years. This trend can be attributed to several factors, such as the wide range of commercial enforcement applications, availability of suitable technologies and good user acceptance [1]. Even if there are several application contexts, in this work, we focus on the recognition of the periocular region dynamics to effectively recognize the subject even if the lower part of the face is covered by a face mask.

Several researchers have tried to investigate and solve this problem [30]. It is worth considering the performance of the typical deep neural network used for recognition decrees when face masks are included in the test set. They increase when masked faces are also included in the training set. In this case, focusing on the periocular region helps to avoid having to deal with the presence of masks in the data. In addition, facial dynamics are an added value that prevents the reduction in spatial information.

## 5. Experimental Analysis

**Figure 2:**
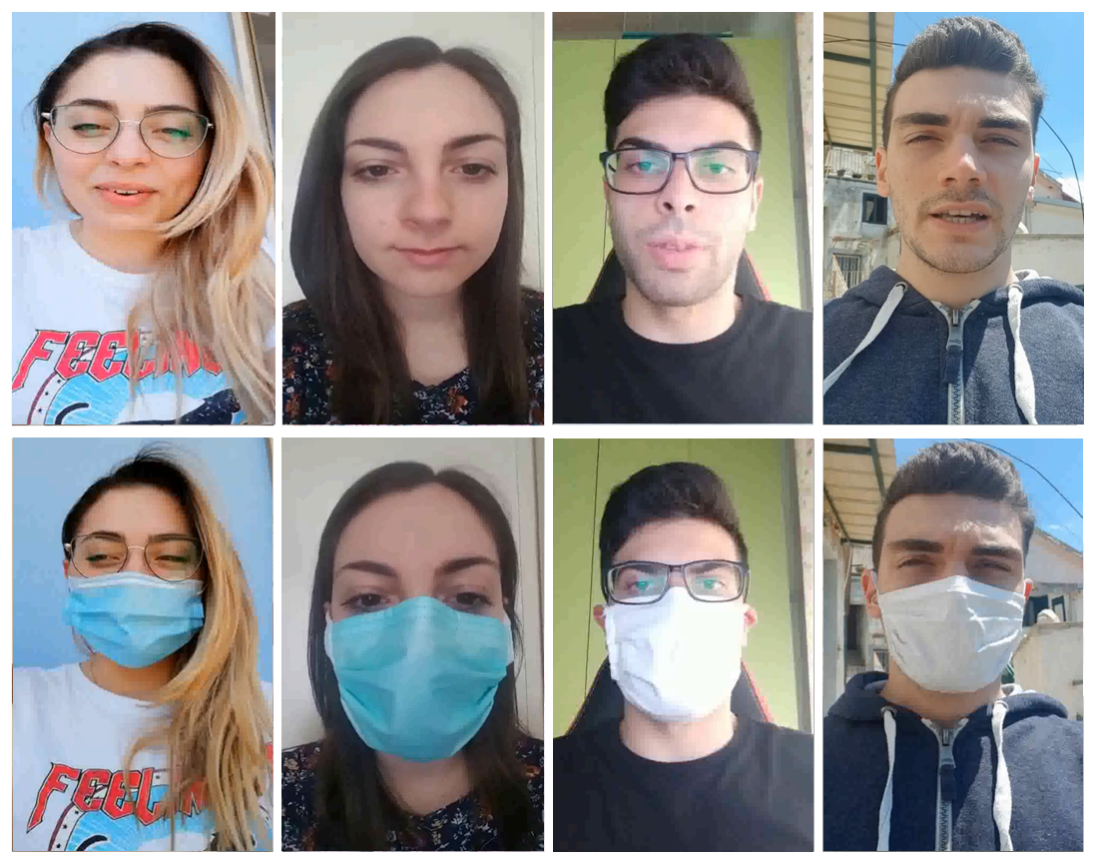
Examples faces with and without masks from the M2FRED dataset.

Face recognition is a biometric technique designed to uniquely identify a person by comparing and analyzing models based on facial characteristics/features. To increase its reliability, the system must be able to distinguish the real face coming from an identifying system under stressful conditions. It has become increasingly evident that the face recognition algorithms offer a truthful performance in a real recognition system. To evaluate how our algorithms work under practical conditions, we have implemented a hybrid facial recognition system of live face detection that uses the sequence analysis of face images captured by a camera as shown in (Figure 2). This system, in addition to allowing for facial recognition with a mask through the processing of SCI, is also able to detect falsification operations by blocking the process.

This counterfeit activity detection, such as the SCI method, works in combination with the face recognition system (Figure 1).

### 5.1. Technical Source Camera Identification (SCI) Information

**Figure 3:**
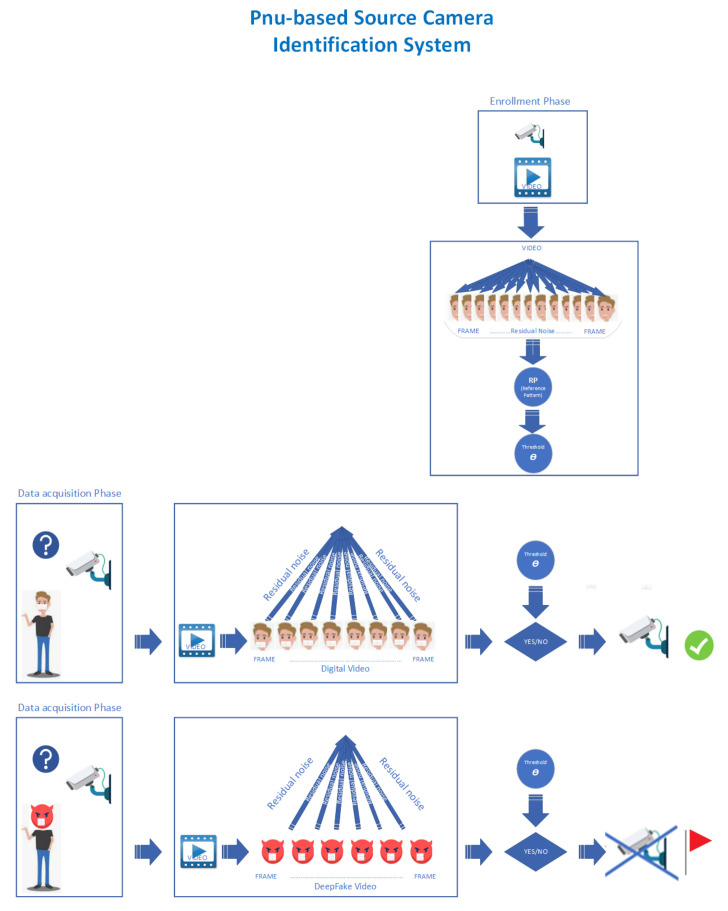
Source Camera Identification Process with a video.

To evaluate the integrity of the recorded video in this face recognition system, we exploit a vanilla implementation of the Fridrich et al. procedure presented in Section 2.1.1. We used the process described in Figure 3.

During the enrollment/registration phase, the selected device records a video. Once the frames have been extracted, the SCI procedure (see Section 2.1) calculates the RP (Reference Pattern) and the threshold θ. In a controlled environment, during the acquisition phase, when the system needs to observe a suspicious subject with a face mask, it will extract the frames from the recorded video and for each of them, it will calculate the RNs (Residual Noises). Using the correlation index (see Equation (3)), it will compare them with the RP generated in the enrollment (registration) phase. We consider the procedure successful if, for all the frames present in the input video, the value of the correlation index is greater than a certain threshold θ [25]. Depending on the value obtained, that is, if the SCI method was successful, the face recognition system continues to work; otherwise, it stops immediately. As shown in Figure 1, just like black boxes, the SCI procedure works to identify counterfeit tasks contextually to the recognition process.

### 5.2. Technical Face Recognition Information

**Figure 4:**
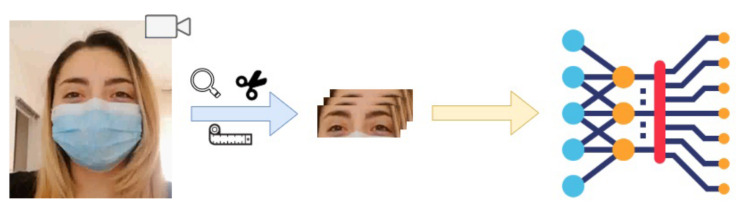
A brief workflow of the face recognition method.

The proposed CNN-LSTM architecture allows to extract the spatial characteristics from each frame and the temporal characteristics between the frames. It is possible to identify two basic steps in the facial recognition process: (1) a pre-processing phase, in which the input videos are processed to obtain the frame sequences of the periocular region and in which the data are standardized, and (2) an extraction step of the characteristics and classification to have a structure that allows us to recognize the faces of the subjects. The overall workflow is shown in Figure 4.

Phase 1

To extract the periocular area, it must first be identified in the image. For this purpose, DLIB, an advanced machine learning library adopted mainly to detect the facial landmarks of an individual, was used. Facial mark detection is defined as a key reference detection activity points on the face (eyes, eyebrows, nose, jaw, mouth, etc.) and then tracing them to determine their spatial coordinates. The system detects the face first, using a face detector, then uses an un-predictor on the face found to obtain the positions. Both the detector, based on the HOG + SVM technique, and the one based on the CNN, are contained in the DLIB library. The predictor in DLIB was used, which detects 68 facial landmarks.

Phase 2

**Figure 5:**
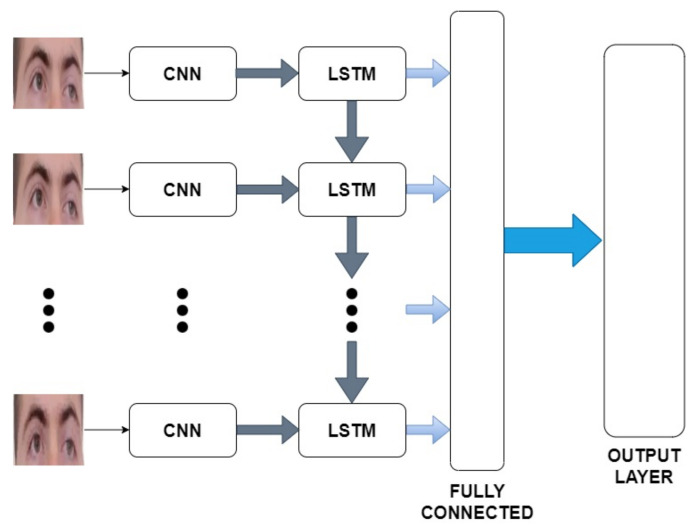
The neural network architecture.

To perform the classification process, and thus recognize objects, a CNN2D-LSTM architecture was used. The proposed model consists of three main modules (Figure 5):

1. A CNN2D structure that receives an image as input and extracts the spatial features and transfers them to the next layer.
2. A LSTM structure that extracts the temporal information related to the video sequence. The recurrent layers are applied to the features extracted in the CNN2D module. The obtained characteristics are sent to the next layer.
3. Fully connected layers for the classification task: this module generalizes the input features giving as a response a label representing the identity of the subject.

The architecture uses a well-known CNN structure: VGG16 [11], for the image features extraction. The latest layers (fully connected, ReLU, and Soft-Max) have been removed from VGG16. A flattened layer is cascaded to the CNN to reduce the dimensionality of the features.The LSTM and Fully Connected layers follow. The VGG16 network used for the features extraction was pre-trained on the VGGFace dataset. It consists of 3.31 million images of 9131 subjects obtained through Google ImageSearch.

## 6. Results and Discussion

To evaluate the performance of the controlled hybrid recognition system, we perform a large-scale experiment using both a dataset that already exists in current literature, as well as a new one.

### 6.1. Datasets

The first dataset is M2FRED (http://biplab.unisa.it/home/m2fred, accessed on 30 May 2022). It was assembled for facial recognition purposes, and it is composed of videos of 46 different subjects, with and without masks, captured indoors and outdoors. For each subject, the video data acquired must be uploaded through a dedicated Google module or via social media.

The above-mentioned acquisition procedure involves the recompression of the video, which causes problems in the SCI method [31]. Then, we assembled a second small dataset, named UntouchedVideo, made up of 48 videos from three distinct devices that register the faces of different subjects, with and without masks, captured in internal and external environments. These videos were downloaded directly to the storage computer without any further compression. We also considered the problem of the *“alien frame”*, that is a frame captured by a specific camera (or device) and then injected into a recorded video using a different camera. Current literature [25] discusses how it is possible to detect the inclusion of alien frames in standard videos (i.e., no deep fake technology applied). We aim to demonstrate that, in the context of videos, this approach is still capable of detecting altered frames with deep fake techniques. Therefore, we have assembled a small deep fake video set consisting of six deep fakes generated from the videos of different subjects belonging to the M2FRED dataset and six deep fakes generated from the second small UntouchedVideo dataset.

We used the DeepFaceLab (https://github.com/iperov/DeepFaceLab, accessed on 30 May 2022) library to generate the deep fake videos.

### 6.2. Part A: Evaluating the Performance of the Face Recognition Method

To evaluate the performance related to the applied model, metrics such as accuracy, precision, recall, and f1-score are calculated. Confusion Matrix and ROC curves are also considered to assess the classification errors.

**Figure 6:**
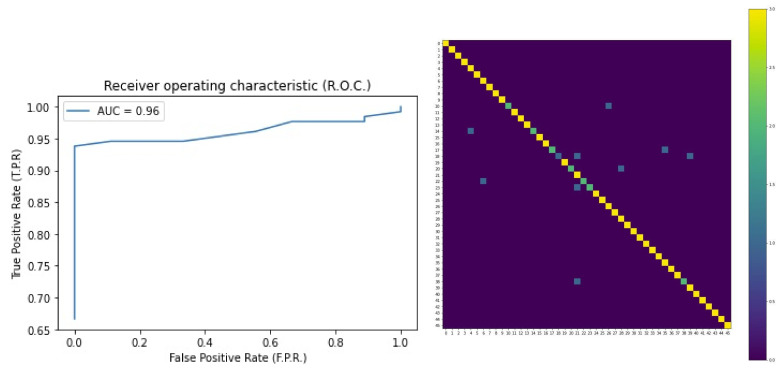
ROC Curve (**left**) and Confusion Matrix (**right**) for the face recognition module on the M2FRED dataset.

The results obtained by applying the CNN-LSTM model described in Section 5.2 are shown in Table 1. It can be noted that this model, applied to masked subjects belonging to the M2FRED dataset, achieves a precision of 0.96 and a recall, f1-score, and accuracy of 0.95. In Figure 6, the ROC curves and the Confusion Matrix show good behavior of the method for the classification task.

The trained model was also applied to the six deep fake videos generated from the M2FRED dataset. Our goal was also to check whether a simple facial recognition system is reliable when processing tampered videos.

In Table 2, the results show that for each deep fake, the system classifies the subjects based on the *Source*: this means the tampered videos can fool the facial recognition system. The face superimposed on the video of subject 038 for deep fake 5 is classified as 027 rather than 036; it is worth noting how, in this particular case, the prediction score is very low. In the remaining cases, the predicted identities are always correctly associated with the actual identities of the *Sources*. The classification scores for each tempered video are also shown in Table 2. In most cases, the probability for each classified subject is higher than 50%.

In this part of the experimentation, the most significant result highlighted that the facial recognition system is not able to distinguish an original video from a tampered one. Consequently, it was necessary to add a level of security to the biometric system.

### 6.3. Part B: Evaluating the Performance of the SCI Approach

We experimented to assess if the SCI technique described in Section 2.1 can detect forgery operations in a biometric facial recognition system.

In the first step of the experiment, we considered the UntouchedVideo dataset in which no artifacts were applied except video compression. With an estimated statistical correlation with the Neyman–Pearson method (see Equation (3)), we measured the similarity between the Residual Noise RN of each frame of the video under scrutiny and the Reference Pattern RP of its digital sensor.

Then we calculated a specific threshold θ for each device, represented by the value average correlation index between the RP of the device that recorded the video and the RN of the respective frames.

**Figure 7:**
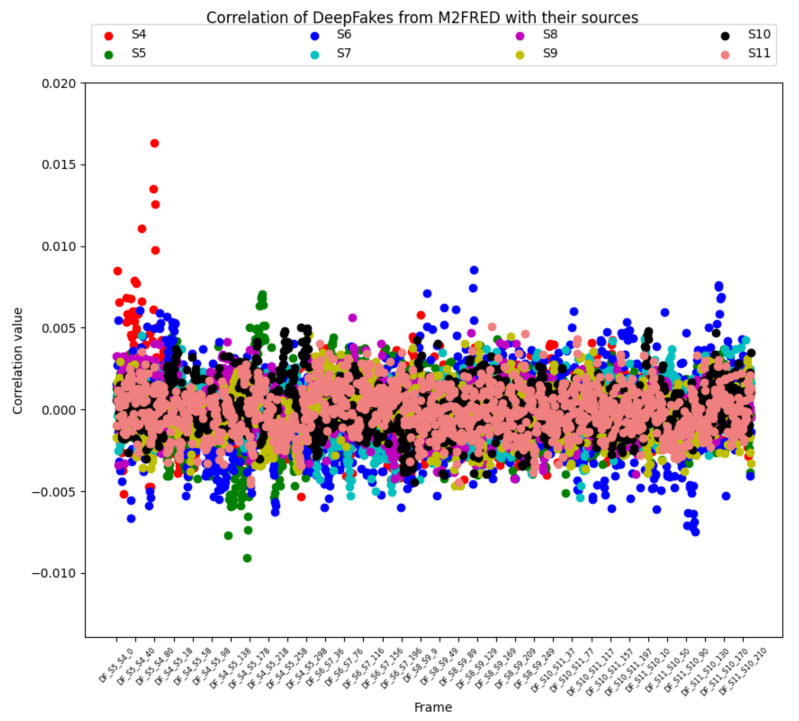
Correlation value between deep fake videos generated from the M2FRED dataset and their sources.

In the second step of the experiment, for completeness, we performed an experiment on six videos generated from the M2FRED dataset, but for the above-mentioned compression problem, we obtained inconclusive results, as shown in Figure 7. Graphically the correlation index values, calculated between the RP of a selected device, and the RNs of the frames coming from the video recorded by that device, fluctuate above and below the red line of the recognition threshold θ. In practice, the manipulations (i.e., recompression) apply a bias, making it no longer recognizable. We came to the conclusion that in this specific case, where the videos were uploaded via a dedicated Google module or via social media, the SCI method does not work. Finally, we repeated the experiment on another six videos generated from the UntouchedVideo dataset (see Table 3).

We have measured the similarity between the RPs and the RNs values as in the previous step and for each device the results were compared with the aforementioned recognition threshold. Whenever the correlation index between the RP and RN did not exceed the θ threshold, then it was highly probable that the frame was unreliable. In this case, the SCI method has identified possible alterations of the video under scrutiny causing the failure of a manipulation attempt to pass the controls.

**Figure 8:**
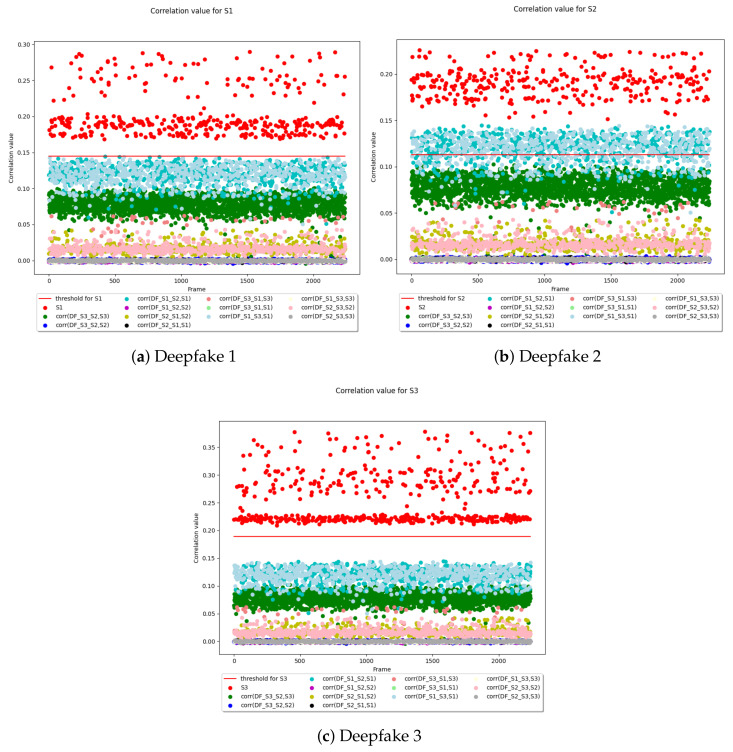
In each sub-figure, (1) the threshold θ, represented by a red line, and (2) the correlation between the RP devices and the RN videos are represented for each of the three devices. The points are plotted considering the original frame position in the videos (*x*-axis) and the correlation value (*y*-axis) between the RN (Residual Noise) extracted from the frame and the RP (Reference Pattern) of the camera under scrutiny.

Figure 8 graphically presents the recognition threshold θ for each of the three devices, taking into account the correlation between the RP devices and the RN videos. In all the cases of deep fake videos, the aforementioned threshold θ was never exceeded by all the frames of a video, this means that the SCI procedure recognized the videos. On the contrary, the correlation index values, colored in red, between the RP of a device that recorded the video and the RNs of the respective frames are always all above the red threshold θ.

For example, in Figure 8a, all the correlations carried out have been performed against the RP of camera S1. The experiment confirmed that all the correlation values of the RN from unmodified frames are positioned above the red line (the red points) when related to the source camera. All the other correlation values are always below the threshold θ because either the frames come from an artificial video (produced by AI) or the frames have been produced by a different camera.

### 6.4. Hybrid PNU-Based Facial Recognition Architecture

**Figure 9:**
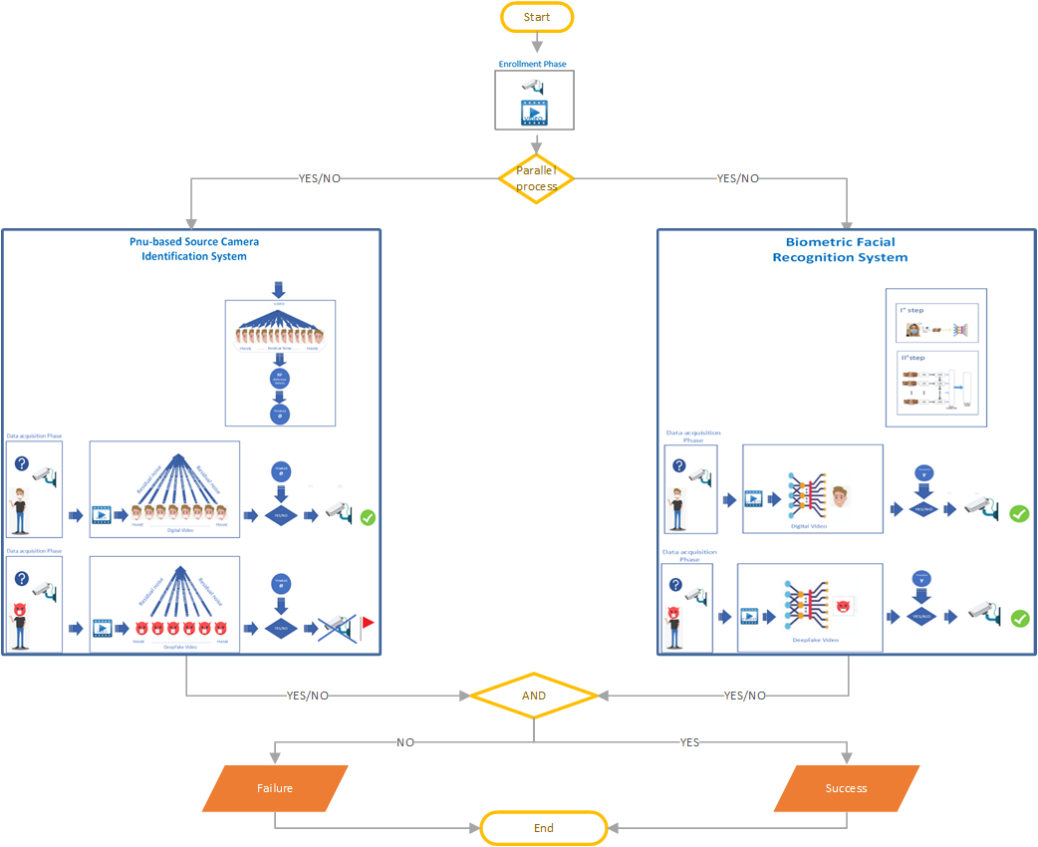
Hybrid PNU-based facial recognition architecture: the left side represents the Pnu-based SCI method and the right side depicts a facial recognition system.

To make a biometric recognition system robust under conditions where a subject is wearing a face mask/covering or in the presence of attempted fraud, we have designed a system in which the facial recognition process and the SCI method work in combination (see Figure 1 and Figure 9). Consequently, a spoof attack is more unlikely to falsify a system based on two techniques rather than one. At the end of our experiments, we have shown that, even when the facial recognition system fails due to forgery operations, the SCI method recognizes the counterfeit, allowing for the success of the hybrid biometric system.

## 7. Conclusions and Future Directions

In this paper, we wanted to focus on finding a methodology to improve face recognition systems with when wearing face masks/coverings in cases and an attempt to commit fraud is possible. Accordingly, we presented a hybrid biometric system considering two types of activities: (i) the facial recognition from video and (ii) the acquisition device used to acquire digital images and their integrity.

We analyzed the methods used for each activity, highlighting the need to comply with the starting requirements. First, for the success of the entire hybrid recognition system presented, it should be emphasized both the rigor with which the initial data must be acquired during the registration phase and the availability of the device (or images captured by the camera) in question. Starting from these assumptions, we conducted a large-scale-experiment to determine the performance of the two different methods for robust facial recognition, working on two datasets of different sizes, summarizing and discussing the results. We also verified that, in cases where the biometric facial system fails, particularly in the cases of videos, the SCI method can detect any manipulations.

The results highlight how, in real-world scenarios, such as automated crowd surveillance, access control, and restricted areas, identification of criminals, law enforcement applications, and so on, the solution offered by the combination of the two methodologies, which are (a) the process of identifying a device and image integrity and (b) the proposed facial recognition system, improves the reliability of biometric recognition systems. To improve the temporal performance, we are planning to carry out a feasibility study on a biometric facial recognition system that combines both single systems in a distributed hybrid approach by using an Apache-Spark system on SCI.

## Figures and Tables

**Table 1:** Accuracy, precision, recall, and f1-score metrics for the face recognition system applied to the M2FRED dataset.

	Precision	Recall	f1-Score	Accuracy
macro-avg	0.96	0.95	0.95	
weighted-avg	0.96	0.95	0.95	
				0.95

**Table 2:** The deep fake videos have two components: the *Source* video, which represents the identity of the subject’s face applied to another video, and the *Destination*, which is the video on which the fake face is applied.

	Source	Destination	Predicted	Score
**deep-1**	001	000	001	0.72
**deep-2**	000	001	000	0.58
**deep-3**	009	010	009	0.87
**deep-4**	013	016	013	0.49
**deep-5**	036	038	027	0.09
**deep-6**	038	036	038	0.83

**Table 3:** Source: name of the devices used for the UntouchedVideo dataset. Device: device model; DeepFake1 and DeepFake2 are the videos generated from the UntouchedVideo dataset. They are obtained by superimposing the face of a selected video (*Destination*) on the face of the original one (*Source*).

Source	Device	DeepFake1	DeepFake2
S1	iPhone 12 Pro	DF_S1_S2	DF_S1_S3
S2	iPhone XS	DF_S2_S1	DF_S2_S3
S3	iPhone 11	DF_S3_S1	DF_S3_S2

## Data Availability

The M2FRED dataset that supports the findings of this study is available at http://biplab.unisa.it/home/m2fred.

## References

[1] Zhao W., Chellappa R., Phillips P.J., Rosenfeld A. (2003). Face Recognition: A Literature Survey. ACM Comput. Surv..

[2] Damer N., Grebe J.H., Chen C., Boutros F., Kirchbuchner F., Kuijper A. The Effect of Wearing a Mask on Face Recognition Performance: An Exploratory Study. Proceedings of the 2020 International Conference of the Biometrics Special Interest Group (BIOSIG).

[3] Damer N., Boutros F., Süßmilch M., Kirchbuchner F., Kuijper A. (2021). Extended evaluation of the effect of real and simulated masks on face recognition performance. IET Biom..

[4] Kumar R.S., Rajendran A., Amrutha V., Raghu G.T. (2021). Deep Learning Model for Face Mask Based Attendance System in the Era of the COVID-19 Pandemic. Proceedings of the 2021 7th International Conference on Advanced Computing and Communication Systems (ICACCS).

[5] Proença H., Neves J.C. (2018). A reminiscence of “Mastermind”: Iris/periocular biometrics by “In-Set” CNN Iterative analysis. IEEE Trans. Inf. Forensics Secur..

[6] Ullah N., Javed A., Ghazanfar M.A., Alsufyani A., Bourouis S. (2022). A novel DeepMaskNet model for face mask detection and masked facial recognition. J. King Saud Univ. Comput. Inf. Sci..

[7] Abdelwhab A., Viriri S. (2018). A survey on soft biometrics for human identification. Mach. Learn. Biom..

[8] Zewail R., Elsafi A., Saeb M., Hamdy N. (2004). Soft and hard biometrics fusion for improved identity verification. Proceedings of the The 2004 47th Midwest Symposium on Circuits and Systems, 2004, MWSCAS’04.

[9] Abate A.F., Barra P., Barra S., Molinari C., Nappi M., Narducci F. (2019). Clustering facial attributes: Narrowing the path from soft to hard biometrics. IEEE Access.

[10] Schroff F., Kalenichenko D., Philbin J. Facenet: A unified embedding for face recognition and clustering. Proceedings of the IEEE Conference on Computer Vision and Pattern Recognition.

[11] Simonyan K., Zisserman A. (2014). Very Deep Convolutional Networks for Large-Scale Image Recognition. arXiv.

[12] Levada A.L.M., Correa D.C., Salvadeo D.H.P., Saito J.H., Mascarenhas N.D.A. Novel approaches for face recognition: Template-matching using Dynamic Time Warping and LSTM neural network supervised classification. Proceedings of the 2008 15th International Conference on Systems, Signals and Image Processing.

[13] Tsai M., Wu G. USING Image Features to Identify Camera Sources. Proceedings of the 2006 IEEE International Conference on Acoustics Speech and Signal Processing Proceedings.

[14] Lukas J., Fridrich J., Goljan M. (2006). Digital camera identification from sensor pattern noise. IEEE Trans. Inf. Forensics Secur..

[15] Kang X., Li Y., Qu Z., Huang J. (2012). Enhancing Source Camera Identification Performance With a Camera Reference Phase Sensor Pattern Noise. IEEE Trans. Inf. Forensics Secur..

[16] Goljan M. (2008). Digital camera identification from images–estimating false acceptance probability. Proceedings of the International Workshop on Digital Watermarking.

[17] Moghaddam B., Pentland A. (1997). Probabilistic visual learning for object representation. IEEE Trans. Pattern Anal. Mach. Intell..

[18] Wiskott L., Krüger N., Kuiger N., Von Der Malsburg C. (1997). Face recognition by elastic bunch graph matching. IEEE Trans. Pattern Anal. Mach. Intell..

[19] Turk M., Pentland A. (1991). Eigenfaces for recognition. J. Cogn. Neurosci..

[20] Abate A., Cimmino L., Nappi M., Narducci F. (2022). Fusion of Periocular Deep Features in a Dual-Input CNN for Biometric Recognition. Proceedings of the Image Analysis and Processing—ICIAP 2022.

[21] Li B., Chellappa R. (2001). Face verification through tracking facial features. JOSA A.

[22] Baccouche M., Mamalet F., Wolf C., Garcia C., Baskurt A. (2010). Action classification in soccer videos with long short-term memory recurrent neural networks. Proceedings of the International Conference on Artificial Neural Networks.

[23] Iengo D., Nappi M., Ricciardi S., Vanore D. Dynamic Facial Features for Inherently Safer Face Recognition. Proceedings of the 2019 IEEE International Conference on Image Processing (ICIP).

[24] Abate A.F., Cimmino L., Narducci F., Pero C. Biometric Face Recognition Based on Landmark Dynamics. Proceedings of the 2020 IEEE Intl Conf on Dependable, Autonomic and Secure Computing, Intl Conf on Pervasive Intelligence and Computing, Intl Conf on Cloud and Big Data Computing, Intl Conf on Cyber Science and Technology Congress (DASC/PiCom/CBDCom/CyberSciTech).

[25] Cattaneo G., Roscigno G., Bruno A., Blanc-Talon J., Distante C., Philips W., Popescu D., Scheunders P. (2016). Using PNU-Based Techniques to Detect Alien Frames in Videos. Proceedings of the Advanced Concepts for Intelligent Vision Systems.

[26] Chen M., Fridrich J., Goljan M., Lukas J. (2008). Determining Image Origin and Integrity Using Sensor Noise. IEEE Trans. Inf. Forensics Secur..

[27] Bruno A., Cattaneo G., Ferraro Petrillo U., Capasso P. (2021). PNU Spoofing: A menace for biometrics authentication systems?. Pattern Recognit. Lett..

[28] Bayram S., Sencar H.T., Memon N. Efficient techniques for sensor fingerprint matching in large image and video databases. Proceedings of the Media Forensics and Security II, International Society for Optics and Photonics.

[29] Chen M., Fridrich J., Goljan M., Lukáš J. Source digital camcorder identification using sensor photo response non-uniformity. Proceedings of the Security, Steganography, and Watermarking of Multimedia Contents IX, International Society for Optics and Photonics.

[30] Jeevan G., Zacharias G.C., Nair M.S., Rajan J. (2022). An empirical study of the impact of masks on face recognition. Pattern Recognit..

[31] Bruno A., Cattaneo G. (2020). An experimental estimate of the impact produced on PNU by new generation video codecs. Int. J. Embed. Syst..

